# Accumulation and deposition of triacylglycerols in the starchy endosperm of wheat grain

**DOI:** 10.1016/j.jcs.2021.103167

**Published:** 2021-03

**Authors:** Irene González-Thuillier, Till K. Pellny, Paola Tosi, Rowan A.C. Mitchell, Richard Haslam, Peter R. Shewry

**Affiliations:** a Rothamsted Research, Harpenden, Hertfordshire, AL5 2JQ, UK; b School of Agriculture, Policy and Development, University of Reading, Whiteknights Campus, Early Gate, RG6 6AR, UK

**Keywords:** Wheat, Starchy endosperm, White flour, Oil, Triacylglycerol, Oleosin

## Abstract

A combination of lipidomics, transcriptomics and bioimaging has been used to study triacylglycerol synthesis and deposition in the developing starchy endosperm of wheat. The content of TAG increased between 14 and 34 days after anthesis, from 50 to 115 mg/100 g dry wt and from about 35 to 175 mg/100 g dry wt in two experiments. The major fatty acids were C16 (palmitic C16:0 and palmitoleic C16:1) and C18 (stearic C18:0, oleic C18:1, linoleic C18:2 and linolenic C18:3), with unsaturated fatty acids accounting for about 75–80% of the total throughout development. Linoleic acid (C18:2) was the major component at all stages and the proportion increased during development. Transcript profiling indicated that predominant route to TAG synthesis and oil accumulation is via the Kennedy pathway and diacylglycerol acyltransferase (DGAT) activity. Confocal microscopy of stained tissue sections showed that TAG accumulated in droplets which are associated with protein and concentrated in the starchy endosperm cells below the sub-aleurone cells. Transcripts encoding 16kd oleosins were also expressed, indicating that the oil droplets are in part stabilised by oleosin proteins.

## Highlights

• Integrated study of triacylglycerol synthesis and deposition in wheat endosperm.
• First lipidomic analysis of triacylglycerol composition in wheat starchy endosperm.
• Bioimaging of lipid deposition in starchy endosperm cells.
• Transcriptome analysis shows operation of Kennedy pathway for lipid synthesis.

## Graphical abstract

**Figure:**
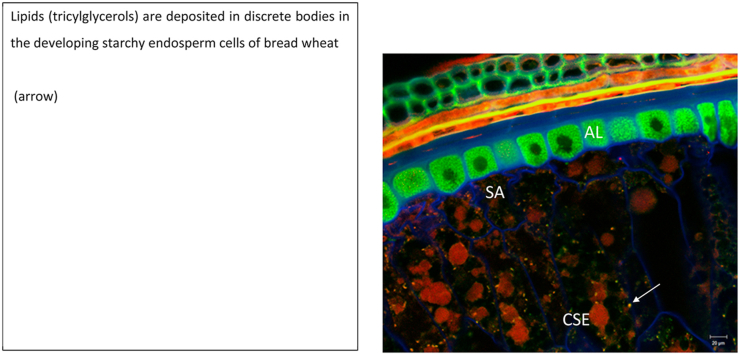

## 1 Introduction

Wheat is the dominant crop and major staple food in Europe, North Africa, West and Central Asia and North and South America, where it contributes between 20% and 50% of the total calories in the human diet. Furthermore, the consumption of wheat is also increasing in countries where it is not readily grown, particularly Sub-Saharan Africa. The global success of wheat is due to its wide adaptability and to the grain processing properties, in particular the ability of wheat flour to be processed into bread, other baked products, pasta and noodles.

The processing properties of wheat are largely determined by the gluten proteins which interact to form a continuous viscoelastic network in dough: this provides the cohesion required for making pasta and noodles, and enables the entrapment of carbon dioxide released during proofing to give the light porous crumb structure of leavened bread. Consequently, wheat proteins have been widely studied. However, gluten proteins are not the sole determinant of processing quality and other grain components also contribute, including starch, cell wall polysaccharides and lipids.

Wheat lipids are typically minor components of grain, accounting for only 2.5–3.3% of whole grain and 2.6–2.7% of white flour (Chung et al., 2009). Nevertheless, they affect the volume and texture of loaves and other baked products (Pycarelle et al., 2019), probably by a combination of effects including binding to and plasticising the gluten network and stabilizing the gas cells which are formed during dough mixing and expanded during fermentation (Salt et al., 2018).

Wheat grain lipids display wide structural diversity, with over 70 molecular species being identified, and comprise neutral (acylglycerols and free fatty acids) and polar (glycolipids and phospholipids) components. Polar lipids are structural components of membranes, with the galactolipids being characteristic of the membranes of the amyloplasts (modified plastids), which contain the starch granules (Haschke et al., 1990). Recent studies have focused on surface-active galactolipids which are present in the air-water interface surrounding the gas bubbles in dough and may contribute to their stability (Schaffarczyk et al., 2014; Salt et al., 2018; Melis et al., 2020; Min et al., 2020).

Triacylglycerols (TAGs) are the major storage lipids in seeds but, with the exception of oats, are minor components in cultivated cereals. They are concentrated in the aleurone layer and scutellum of the embryo of wheat, where they account for 60–80% of the total lipids in these tissues (Chung et al., 2009) and are located in discrete oil bodies. By contrast, although TAGs account for about a third of the total lipids in the starchy endosperm tissue from which white flour is produced (Chung al., 2009; Gonzalez-Thuillier et al., 2015), little is known about their synthesis and deposition. Although lipid droplets have been reported in starchy endosperm cells (Hargin et al., 1980), it has also been suggested that some transfer of lipids (including TAGs) from the aleurone and embryo to the flour occurs during milling (Morrison, 1994).

The production of fatty acids and the synthesis of TAGs in plants is a complex process that involves multiple cellular organelles. Fatty acids are synthesised in the plastid by a Type II fatty acid synthase complex. A repeated series of condensation, reduction and dehydration reactions then adds two carbon units to the extending fatty acid chain. The final products of these reactions are fatty acids typically 16 or 18 carbons (C16 and C18) long and attached to an acyl-carrier protein (ACP). While in the plastid a double bond can be introduced through the action of a fatty acid Δ9-desaturase. The ACP moeity is removed by thioesterases and the fatty acids produced in the plastid are then exported to the cytosol, converted to CoA forms and rapidly incorporated into phosphatidylcholine (PC). Further modification by additional desaturation (via fatty acid desaturases, FADs) (see Hajiahmadi et al., 2020 for a detailed description of wheat FADs) or incorporation of functional groups can then occur. The lipids of wheat grains typically contain C16 and C18 fatty acids, notably palmitic acid (C16:0), palmitoleic acid (C16:1), stearic acid (C18:0), oleic acid (C18:1), linoleic acid (C18:2) and linolenic acid (C18:3). A process of acyl editing exchanges fatty acids between PC and the acyl-CoA pool. Once located in the endoplasmic reticulum (ER), fatty acids are assembled into TAG by a combination of two pathways (Supplementary Figure S1). The acyl-CoA-dependent Kennedy pathway begins with the sequential acylation of glycerol-3-phosphate by glycerol-3-phosphate acyltransferases (GPATs) and lysophosphatidic acid acyltransferases (LPAATs) using acyl-CoA to produce phosphatidic acid (PA). This PA can then be dephosphorylated by PA phosphatases to create *de novo* diacylglycerol (DAG). The DAG is then available for two different acyltransferase reactions: diacylglycerol acyltransferases (DGAT) transfer acyl-CoAs to the sn-3 position of DAG to produce TAG; alternatively phospholipid:diacylglycerol acyltransferases (PDAT) transfers the sn-2 acyl group of from phospholipids to DAG, forming TAG (see Li-Beisson et al., 2013 for a detailed description). The contributions of DGAT and PDAT to TAG synthesis are known to vary between species.

We report here the first detailed study of TAG accumulation and deposition in the wheat starchy endosperm during the major grain filling period. This is based on the analysis of hand-dissected tissues to avoid lipid transfer between tissues with the lipid profiles being combined with transcript analysis and confocal microscope imaging of tissue sections. This study therefore add to our currently limited knowledge of the synthesis and deposition of TAG in the starchy endosperm during grain maturation and provides a basis for determining the contributions of TAGs to flour processing and breadmaking.

## 2 Experimental

### 2.1 Plant material

Bread wheat cultivar Hereward (a breadmaking type with hard endosperm texture) was grown in field trials with three replicate blocks in 2016 (year 1) and 2017 (year 2) at Rothamsted Research (Harpenden, UK) for lipidomics and microscopy. For transcript analysis cv Yumai 34 was grown in a glass house with 20 °C/15 °C day/night cycles and a photoperiod of 16 h, supplementary lighting being provided when ambient levels fell below 400 μmol m^−2^ s^−1^. Heads were tagged at anthesis and caryopses harvested from the middle thirds of ears for each developmental stage (14, 21, 28 and 34/5 days post-anthesis (dpa)). Starchy endosperm tissue was dissected by hand and frozen in liquid nitrogen for lipidomics or transcript analysis. Lipid analysis was carried out on three replicate samples of twelve caryopses each, representing the outer fully developing caryopses of the central six florets of one ear. For transcriptomics, caryopses from two or three individual ears were pooled for each sample with two replicates per time point. Whole caryopses were used directly for microscopy.

### 2.2 Lipid extraction

Samples were transferred to a glass tube with 1 mL of propan-2-ol. Samples were crushed with a glass rod, vortexed and then heated at 75 °C for 20min. Chloroform, methanol and H_2_O (1:1:0.7) were then added and the mixture vortexed followed by 1 mL chloroform and 1 mL water and the mixture vortexed again. Two phases were separated by centrifugation and the lower phase, containing the lipids, was removed to a new tube. The extraction was repeated with an additional 1 mL of chloroform and, after mixing and centrifugation, the lower phase was removed to the same tube. The samples were dried with a current of nitrogen and re-suspended in 200 μl chloroform and stored at −80 °C(Bligh and Dyer 1959; Kates et al., 1986).

### 2.3 TAG analysis

Triacylglycerols were identified and quantify by ESI-MS/MS as described by Li et al. (2014) with modifications (Gonzalez-Thuillier et al., 2015). A portion of lipid extract (10 μL) and 0.857 nmol tri15:0-TAG (Nu-Chek Prep, Minnesota, USA) were mixed with chloroform:methanol:300 mM ammonium acetate (24:24:1.75: v/v) to a final volume of 1 ml for direct infusion into the mass spectrometer. TAG was detected as [M + NH4]+ ions by a series of different neutral loss scans, targeting losses of fatty acids. The data were processed using the program Lipid View Software (AB-Sciex, Massachusetts, USA) where isotope corrections are applied. The peak area of each lipid was normalized to the internal standard and further normalized to the weight of the initial sample.

### 2.4 Sample preparation and staining for imaging

Developing caryopses were hand-dissected and immediately fixed in 4% (w/v) paraformaldehyde in 1x phosphate buffer solution (PBS) for 4 or 5 h after removal of the two ends to facilitate penetration of the fixative in the tissue. The samples were then washed x 3 with PBS and cut into 150 μm transverse sections using a Vibratome (Leica VT1000S, Germany). Sections were collected with a fine brush and washed briefly in PBS, before being subjected to two additional hours fixation in fresh 4% (w/v) paraformaldehyde in PBS, following which they were washed three times with PBS for 5–10 min. For confocal microscopy the sections were sequentially stained with BODIPY 493/503 (Thermo Fisher Scientific) for neutral lipids, calcofluor white for cell walls and rhodamine for proteins. The sections were submerged for 2 min in BODIPY solution (1 μg BODIPY per mL of PBS) then washed for 5 min with PBS, 1 min in distilled water and 1 min in PBS. They were then stained with 0.05% calcofluor white for 30 s and washed as above followed by 0.5 μg rhodamine in 1 ml of PBS and washed again. The stained sections were mounted on a slide in fluorescence mounting medium (CITIFLUOR AF1, England) and observed by confocal microscopy (Zeiss LSM780, Germany). Images were acquired with Zen 2011 software.

### 2.5 Transcript analysis

Transcript profiles were determined essentially as described by Pellny et al. (2012) for cv Cadenza. In short, central samples of starchy endosperm were dissected by hand, snap frozen in liquid nitrogen and total RNA extracted using a CTAB method. Transcriptome analysis was performed at the University of Bristol Transcriptomics facilities using Illumina single reads.

## 3 Results and discussion

### 3.1 TAG accumulation

**Fig. 1:**
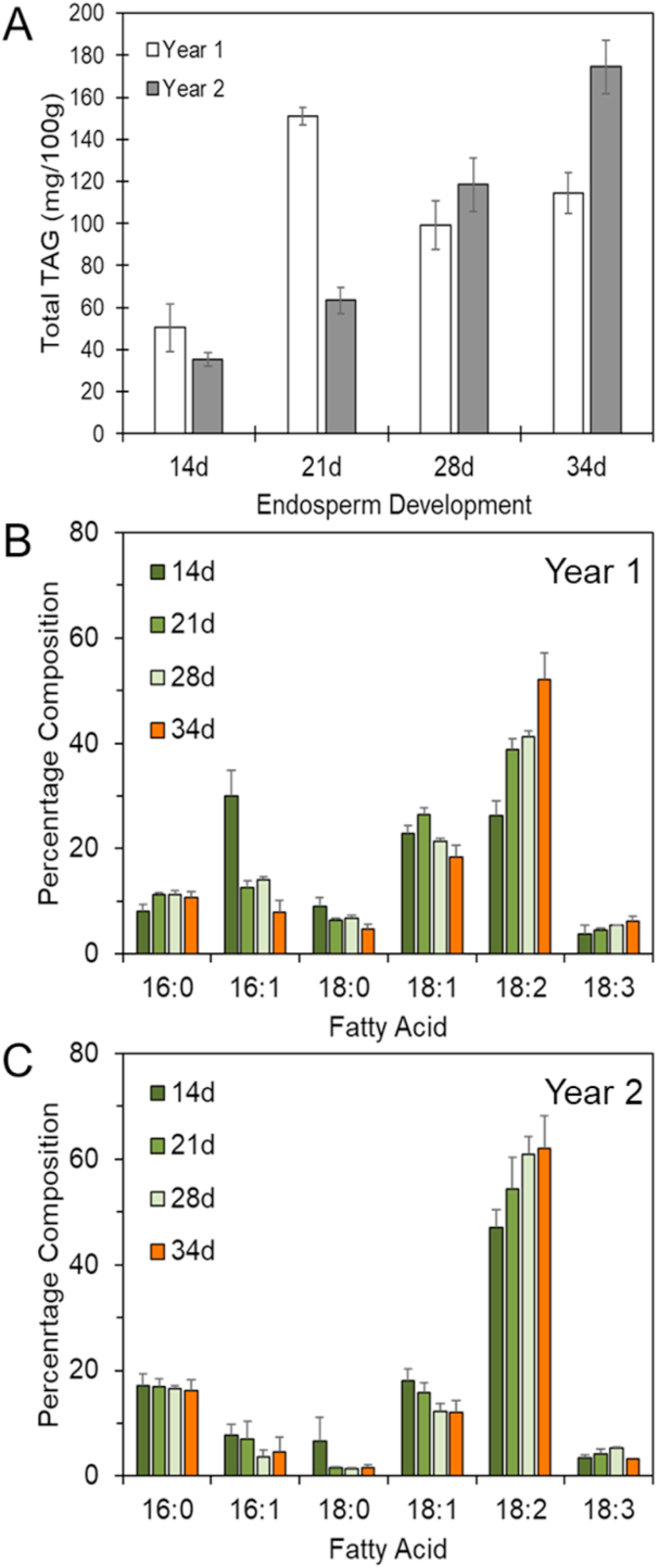
Total triacylglycerols (TAGs) (A) and percentage compositions of fatty acids in TAGs (B,C) in developing endosperm of wheat grown in Years 1 (A,B) and 2 (A,C). Data presented represent the analysis of three hand dissected samples of caryopses+/-standard error Fatty acids are: C16:0, palmitic; C16:1, palmitoleic; C18:0, stearic; C18:1, oleic; C18:2, linoleic; C18:3, linolenic. d, days post-anthesis.

**Fig. 2:**
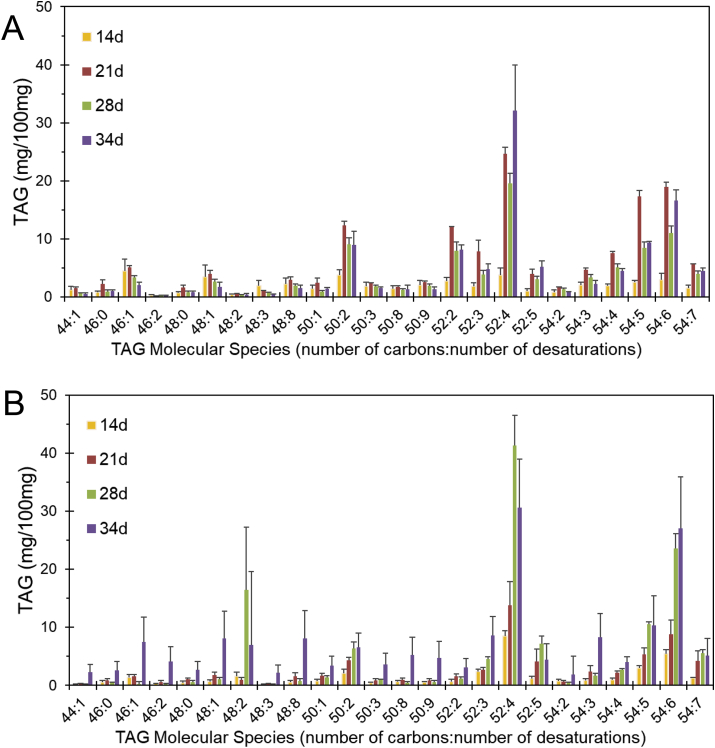
Triacylglycerol (TAG) molecular species in developing starchy endosperm of wheat grown in Years 1 (A) and 2 (B). Data presented represents the analysis of three hand dissected samples of caryopses+/-standard error Molecular species are defined as the sums of carbon atoms (44–54) and double bonds (0–7) in the three fatty acid moieties. d, days post-anthesis

Starchy endosperm tissue was prepared from developing caryopses of wheat cv Hereward between 14 and 34 days post-anthesis (dpa) which correspond to the most active phase of grain filling. Total TAGs and TAG molecular species were determined by lipidomic profiling as described by Min et al. (2017). The experiment was carried out in two years, 2016 and 2017 (called Years 1 and 2, respectively), and the results are summarised in Fig. 1 and Fig. 2

The content of TAG increased during development from about 50 to 115 mg/100 g dry wt in Year 1 and from about 35 to 175 mg/100 g dry wt in Year 2 (Fig. 1A). The fatty acid profiles of the total TAG fractions were broadly similar over the two experiments, although the precise proportions of the fatty acids differed (Fig. 1B and C): this almost certainly resulted from differences in the growth conditions, which are known to strongly affect grain lipid composition (Salt et al., 2018). The major fatty acids were C16 (palmitic C 16:0 and palmitoleic C16:1) and C18 (stearic C18:0, oleic C18:1, linoleic C18:2 and linolenic C18:3), with unsaturated fatty acids accounting for about 75–80% of the total throughout development. Linoleic acid (C18:2) was the major component at all stages and the proportion increased during development.

Twenty-four TAG species were determined (Fig. 2), including two minor saturated species (C46:0, C48:0). The major species in both years was C52:4, but the proportions of all species varied with no consistent trends between stages and years. The accumulation of TAG during endosperm development aligns with its role as a storage reservoir of fatty acids. Furthermore, it is possible that TAG composition is dynamic during development, exchanging fatty acids with cell membranes to modulate membrane properties, including adaptation to environmental conditions (de Carvalho and Caramujo, 2018).

### 3.2 Transcript analysis of TAG synthesis

**Fig. 3:**
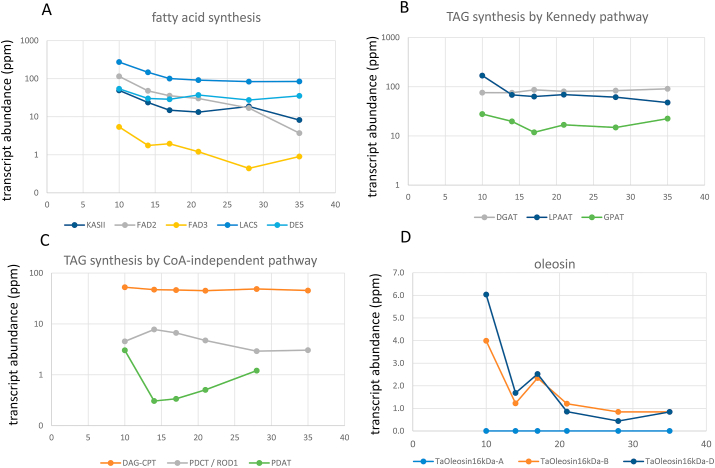
Expression profiles of transcripts encoding enzymes involved in TAG synthesis and oleosins in developing starchy endosperm of wheat between 10 and 35 days post-anthesis (RNAseq data mapped to IWGSC refseq 1.1]). Panel A shows transcript profiles for enzymes associated with fatty acid synthesis:KASII, β-ketoacyl-acyl carrier protein (ACP) synthase II; FAD2, fatty acid desaturase 2; FAD3, fatty acid desaturase 3; LACS, long-chain acyl-CoA synthetase; long-chain acyl-CoA synthetases; DES/SAD, stearoyl-acyl carrier protein-desaturase. Panel B shows acyltransferases catalysing TAG synthesis via the Kennedy pathway: DGAT, diacylglycerol acyltransferases; LPAAT, lysophosphatidic acid acyltransferase; GPAT, glycerol-3-phosphate acyltransferases. Panel C shows enzymes catalysing TAG synthesis via the CoA-independent pathway: DGAT-CTP, diacylglycerol cholinephosphotransferase; PDCT/ROD1, phosphatidylcholine:diacylglycerol cholinephosphotransferase; PDAT, phospholipid:diacylglycerol acyltransferase. Panel D shows transcripts for 16 kDa oleosins (*Ole-1*) encoded by the A, B and D genomes.

The profiles of transcripts encoding enzymes catalysing the synthesis and assembly of TAG were determined using transcript libraries for hand-dissected starchy endosperm samples from the cultivar Yumai 34, harvested at similar developmental stages to those analysed from cv Hereward (Fig. 3).

The transcripts for enzymes catalysing fatty acid synthesis show an initial burst of activity which then continues through grain development (Fig. 3, Panel A). Abundant transcripts include β-ketoacyl-acyl carrier protein (ACP) synthase II (KASII), which elongates 16:0-ACP to 18:0-ACP in the plastid at the first branch point of fatty acid synthesis. At the same time (10–15 DAP) transcripts for acyl-ACP desaturase (stearoyl-acyl carrier protein-desaturase; DES/SAD), which introduces the first double bond into the acyl chain of saturated fatty acid in plastids (C18:0 to C18:1), and FAD2 (which converts C18:1 to C18:2) are also abundant. Long-chain acyl-CoA synthetases (LACSs) typically esterify 16-carbon and mono- and polyunsaturated 18-carbon fatty acids to acyl-CoA and therefore play vital and diverse roles typically associated with cuticular wax synthesis. However, it is not uncommon for LACS mutants to produce less seed oil than wildtype. LACS transcript activity remains high through grain development which is consistent with its established role in supplying substrates for wax biosynthesis and its contribution to TAG assembly (Zhao et al., 2019). The levels of transcripts for FAD3, which converts C18:2 to C18:3, are consistently low, which is consistent with the low proportion of C18:3 in grain lipid profiles (~5%).

Triacylglycerol (TAG) is synthesised by two routes, either in a reaction that uses acyl-CoA as acyl donor and diacylglycerol (DAG) as acceptor (the Kennedy pathway) or from phosphatidyl choline in an acyl-CoA independent reaction (Supplementary Figure S1).

Analysis of the transcript profiles of three acyltransferases involved in the Kennedy pathway (Panel B) shows that the pathway is highly active during grain development, with consistently high levels of transcripts for sn-1 glycerol-3-phosphate acyltransferase (GPAT) which acylates glycerol-3-phosphate to form lysophosphatidic acid (LPA), lysophosphatidic acid acyltransferase (LPAAT) which acylates LPA to give phosphatidic acid and diacylglycerol acyltransferase (DGAT) which catalyses the third acylation, following dephosphorylation of PA, to give diacylglycerol (DAG), to give TAG.

However, transcripts are also present for activities involved in the production of TAG from PC and DAG by the acyl-CoA independent pathway, namely phospholipid:diacylglycerol acyltransferases (PDAT), phosphatidylcholine:diacylglycerol choline phosphotransferase (PDCT) and diacylglycerol cholinephosphotransferase (DAG-CPT). Together with lysophospholipid acyltransferases (LPCAT), these enzymes are responsible for the exchange of DAG and PC head groups and the mixing of acyl-CoA species into the TAG biosynthetic pathway. DAG produced from these exchange activities is often referred to as PC-derived DAG. Hence, both *de novo* synthesised DAG and PC-derived DAG can be used as a substrate for DGAT TAG synthesis. However, the transcript levels for the acyl-CoA independent pathway are generally lower than those for the Kennedy pathway.

The transcript profiles therefore indicate that the predominant route to TAG synthesis and oil accumulation in the developing starchy endosperm of wheat is via the Kennedy pathway and DGAT activity. However, the acyl-CoA independent pathway clearly also operates and the precise contributions of *de novo* DAG (Kennedy pathway) and PC-derived DAG remain to be determined. The importance of the Kennedy pathway is supported by the transcript analysis of wheat grains reported by Grimberg et al. (2020) which also showed low levels of PDAT activity early in grain development and higher levels of DGAT/TAG1 activity.

### 3.3 TAG deposition

Oil bodies in seed tissues are usually stabilised by a surface layer of oleosin proteins associated with phospholipids, with smaller amounts of other proteins (notably, caleosins, LD associated protein and OB associated protein) (Huang, 2018). Oil body proteins have not been identified in white flour or starchy endosperm cells of wheat, although oleosins have been identified in bran, a fraction which contains aleurone cells (Tsen et al., 1990) and embryos (Lv et al., 2016) in addition to the outer tissues of the grain.

**Fig. 4:**
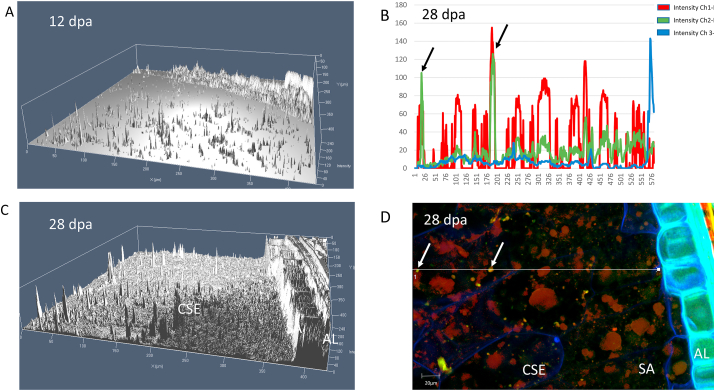
Lipid deposition in the starchy endosperm cells of the developing wheat caryopsis. Panels A and C show three-dimensional representations of the distribution of lipids reconstructed from CLSM images of BODIPY-stained cross sections of developing caryopses at 12 days post-anthesis (dpa) (panel A) and 28 dpa (panel C). Panel B shows the intensity levels of staining with rhodamine (protein), BODIPY (lipids) and calcofluor (cell wall polysaccharides), as detected along the transect showed in panel D. Panel D shows a cross section of a developing caryopsis at 28 dpa wheat grain stained for protein, lipids and cell wall polysaccharides.

We therefore initially used confocal laser microscopy to study the accumulation of oil in the developing starchy endosperm cells of wheat and determine whether the oil deposits were associated with proteins. Staining with BODIPY showed clear droplets of neutral lipids in the starchy endosperm cells of grain sections at 12 and 28 days post-anthesis (Fig. 4D). In order to visually compare the distribution of lipid bodies, confocal images of grain sections at 12 and 28 days post-anthesis are displayed as three-dimensional images in Fig. 4A and C. These show a clear increase in the number of oil deposits between 12 and 28 days. They also show that oil deposits are concentrated in the central starchy endosperm cells (CSE) at both stages, and absent from the sub-aleurone cells (SA). Both sections also show aleurone cells (AL), demonstrating the abundant lipid deposits in this tissue. To determine whether the oil deposits were associated with protein the sections were co-stained for neutral lipids (BIODIPY), protein (rhodamine) and cell wall glucans (β-glucan and cellulose, stained with calcofluor white) (Fig. 4B). The distributions of these components were then determined across transects of the section, running from the outer cell wall of the aleurone layer to the central starchy endosperm. These transects passed through oil deposits, showing that they were clearly associated with peaks in protein concentration (see arrows in Fig. 4B and D).

Oleosins are present in cereal seeds in two isoforms with masses of about 16,000 and 18,000 (16 kDa and 18 kDa oleosins, respectively) (Tsen et al., 1990) and Aalen (1995) reported that transcripts for both were present in the aleurone and starchy endosperm of the barley grain. The expression profiles of transcripts encoding these two forms were therefore determined using the starchy endosperm transcript libraries described above. No transcripts for the 18 kDa oleosins (*Ole-1*) were detected, but transcripts corresponding to the 16 kDa oleosin (*Ole-*2) genes on the B and D (but not A) genomes were detected (Fig. 3D). The *Ole-2* transcipts from the B and D genomes show similar deceases in expression during caryopsis development. These data are therefore consistent with the oil deposits in the starchy endosperm cells being stabilised by 16 KDa oleosins.

### 3.4 General discussion

We have demonstrated that the TAGs deposited in the starchy endosperm cells of developing caryopses are rich in linoleic acid (C18:2), with the proportion of this fatty acid also increasing during caryopsis development. Furthermore, TAGs are deposited in discrete oil deposits which are associated with protein. The presence of *Ole-2* transcripts encoding the 16 kDa oleosin isoform suggests that these bodies are stabilised by oleosins as in most other oil-storing seed tissues. Although we have not directly demonstrated a role of oleosin in stabilizing lipid bodies in this paper, recent studies carried out in transgenic wheat support this suggestion: overexpression of the AsWRI1 transcription factor from oat in the starchy endosperm of wheat resulted in increases in accumulation of TAGs by up to nine-fold and in up-regulation of oleosin-encoding genes (Grimberg et al., 2020). The fact that oleosins have not been reported in published proteomic studies of wheat starchy endosperm or white flour probably reflects technical problems, as oleosins are highly hydrophobic and not readily extracted in buffers used for proteomic studies.

Oats differs from other cereal grain in containing 5–6% oil which is concentrated in the starchy endosperm as well as in the embryo and aleurone. Furthermore, oil is deposited in the sub-aleurone as well as the central starchy endosperm cells. Although an early study using fluorescence microscopy and Nile blue staining to visualise lipids reported the presence of oil bodies (termed spherosomes) in the subaleurone cells of wheat (Hargin et al., 1980), this was not observed in our work, where BODIPY was used as a stain specific for neutral lipids.

Wheat also appears to differ from oats in that the oil deposits in the starchy endosperm remain discrete whereas they merge in the developing oat endosperm, resulting in an oily matrix surrounding the starch and protein in the mature grain (Heenan et al., 2008). This merging of the oil bodies in the starchy endosperm of oats may result from an imbalance between the synthesis of oil and oleosins as Heenen et al. (2008) showed that oleosins are present in all oil-storing tissues of the oat grain, but the amount relative to oil content is much lower in the endosperm (aleurone and starchy endosperm) than in the embryo. In view of the low oil content it is unlikely that a similar merging of oil bodies occurs in the wheat starchy endosperm, but they will certainly become disrupted as the grain matures and the cell contents merge.

## Funding

Rothamsted Research receives strategic funding from the 10.13039/501100000268Biotechnology and Biological Sciences Research Council (BBSRC) and the work forms part of the Designing Future Wheat strategic programme (BB/P016855/1). The work was also supported by the BBSRC Crop Improvement Research Club grant BB/J019526/1 “The role of lipids in determining gas bubble retention in wheat dough”.

## Credit authorship statement

**Irene** **González-Thuillier**: Conceptualization, Methodology, Formal Analysis, Writing- review and editing. **Till K. Pellny:** Formal analysis, Investigation, Methodology, Validation. **Paola Tosi:** Conceptualization, Methodology, Writing - review & editing, Visualization **Rowan Mitchell: Methodology,** Formal analysis, Investigation; Writing- review and editing. **Richard Haslam.** Conceptualization; Methodology, Formal Analysis**;** Supervision, Writing - original draft, Writing - review & editing **Peter R. Shewry:** Conceptualization; Project Administration, Funding acquisition, Supervision, Writing - original draft, Writing - review & editing.

## Declaration of competing interest

The authors declare that they have no known competing financial interests or personal relationships that could have influenced the work reported in this paper.
