# Supplementary material for: Accumulation and deposition of triacylglycerols in the starchy endosperm of wheat grain
Source: J Cereal Sci. 2021 Mar;98:103167. doi: 10.1016/j.jcs.2021.103167 (PMC8047771; doi:10.1016/j.jcs.2021.103167)
Supplement: Multimedia component 1 [file mmc1.pptx]

## Slide 1
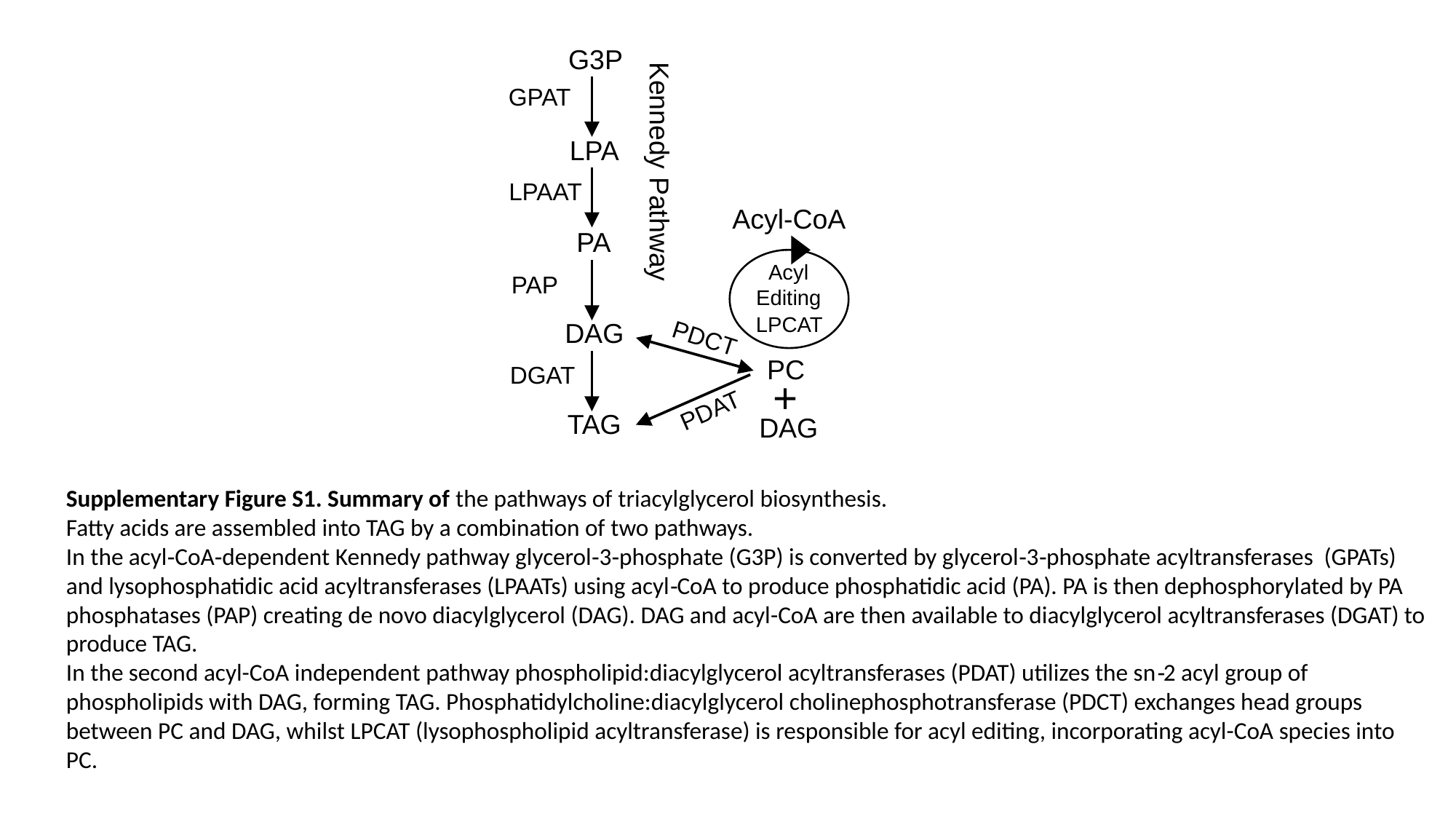

G3P
GPAT
LPA
LPAAT
Acyl-CoA
PA
Acyl
Editing
PAP
LPCAT
DAG
PDCT
PC
DGAT
PDAT
TAG
Kennedy Pathway
+
DAG
Supplementary Figure S1. Summary of the pathways of triacylglycerol biosynthesis.
Fatty acids are assembled into TAG by a combination of two pathways.
In the acyl‐CoA‐dependent Kennedy pathway glycerol‐3‐phosphate (G3P) is converted by glycerol‐3‐phosphate acyltransferases (GPATs) and lysophosphatidic acid acyltransferases (LPAATs) using acyl‐CoA to produce phosphatidic acid (PA). PA is then dephosphorylated by PA phosphatases (PAP) creating de novo diacylglycerol (DAG). DAG and acyl-CoA are then available to diacylglycerol acyltransferases (DGAT) to produce TAG.
In the second acyl-CoA independent pathway phospholipid:diacylglycerol acyltransferases (PDAT) utilizes the sn‐2 acyl group of
phospholipids with DAG, forming TAG. Phosphatidylcholine:diacylglycerol cholinephosphotransferase (PDCT) exchanges head groups between PC and DAG, whilst LPCAT (lysophospholipid acyltransferase) is responsible for acyl editing, incorporating acyl-CoA species into PC.
